# Immunosuppressive impact of *Aedes triseriatus* salivary gland extract on lymphocyte biology

**DOI:** 10.3389/fimmu.2025.1701532

**Published:** 2026-01-27

**Authors:** Molly Ring, Paola Carolina Valenzuela Leon, Brian Bonilla, Jian Wu, Caroline Percopo, Xin-zhuan Su, Eric Calvo

**Affiliations:** Laboratory of Malaria and Vector Research, National Institute of Allergy and Infectious Diseases, National Institutes of Health, Rockville, MD, United States

**Keywords:** arthropods, blood feeding, lymphocyte biology, mosquito, vector-borne diseases

## Abstract

**Background:**

Mosquito salivary proteins play a crucial role in blood meal acquisition and are known to disrupt host immune responses and homeostasis. Previous studies have identified salivary gland extract from the prominent arbovirus vector, *Aedes aegypti*, that contains pharmacologically active proteins which can induce cell death in splenic lymphocyte populations in mice. However, it has remained unclear until now whether this effect is unique to *A. aegypti* or conserved across other *Aedes* species.

**Methods:**

Here, we characterize the immunomodulatory properties of the salivary gland extracts from *Aedes triseriatus*, the primary vector of La Crosse virus (LACV). Murine, guinea pig, and human lymphocytes were exposed to salivary gland extracts at varying concentrations and time points. Lymphocyte proliferation was evaluated using colorimetric metabolic assays and flow cytometry analysis to characterize apoptotic mechanisms and define affected lymphocyte subsets.

**Results:**

We found that *Ae. triseriatus* salivary gland extract inhibited splenocyte proliferation in both mice and guinea pigs through the induction of apoptosis and suppression of cytokine expression. We identified a strong dose-dependent apoptotic phenotype present in CD4^+^, CD8^+^, and CD19^+^ lymphocytes. However, primary human lymphocytes and human lymphoid cell lines did not exhibit reduced proliferation after exposure to salivary gland extracts.

**Conclusion:**

Together, these discoveries suggest that *Aedes species* saliva contains evolutionarily adapted immunomodulatory proteins that could help facilitate arboviral persistence in rodent reservoirs.

## 1 Introduction

Mosquito salivary proteins play a critical role in counteracting host hemostasis and immune defenses during blood meal acquisition. The saliva of mosquitoes contains a complex mix of pharmacologically active proteins that contribute to vasodilation, inhibition of coagulation pathways, reduction of platelet aggregation, and modulation of host immune factors (1–7). A detailed understanding of the function of these salivary proteins is essential not only for clarifying the mechanisms involved in blood meal acquisition but also for identifying how specific salivary components may enhance or interfere with pathogen transmission. Salivary protein can also interact with viruses (8–10), impacting viral transmission and vertebrate immune evasion. In addition, the ability of mosquito saliva to manipulate host immune factors is crucial in the early stages of arbovirus infection. *Aedes* genus mosquitoes have been a primary focus of research as the major vector for arboviruses such as dengue, chikungunya, yellow fever, and Zika viruses (11, 12). As a result, extensive efforts have been made to characterize how their saliva can shape both the innate and adaptive immune responses of their hosts and how these effects can influence arbovirus pathogenesis (10, 13–20).

The innate immune response acts as the first line of defense against pathogens and is driven cellularly by various leukocyte populations that carry out a less antigen-specific, but rapid, immune activation (21) (15, 20, 22). These processes create niches of myeloid cells, facilitating early viral replication. For flaviviruses, distinct salivary proteins have been shown to contribute to pathogenesis. A member of the antigen-5 related proteins, AaVA-1, facilitates dengue and Zika replication in monocytes by modulating autophagy (19), while NeSt1 promotes inflammatory mediators such as IL-1β and CCL2 (23).

The antigen-specific adaptive immune response, heavily driven by T- and B-lymphocytes, is also a target of *A. aegypti* saliva immunomodulation. In murine models, low doses of *A. aegypti* saliva have been shown to strongly inhibit CD4^+^ T cell proliferation and inhibit some antigen-stimulated cytokine production, including Interleukin-2 (IL-2) and interferon gamma (INF-γ), as well as proinflammatory cytokines, tumor necrosis factor alpha (TNF-α), and granulocyte-macrophage colony-stimulating factor (GM-CSF) (24). Saliva from *A. aegypti* has also been shown to induce cell death in murine splenic CD4^+^ and CD8^+^ T cells as well as B cells. A cytotoxic effect was not observed in cells treated with saliva from *Culex* genus mosquitoes (25). Research into the mechanism behind this apoptotic pathway described activation of both caspase-3 and caspase-8 (26). It remains unclear whether this cytotoxic effect is unique to *A. aegypti* or if it is conserved across other *Aedes* species.

Our study attempts to bridge this knowledge gap by characterizing the immunomodulatory activity of salivary gland extracts (SGEs) from *A. triseriatus* mosquitoes. *Aedes triseriatus*, commonly known as the eastern treehole mosquito, is the primary vector for La Crosse virus (LACV) in North America. The La Crosse virus, Orthobunyavirus, is the causative agent of La Crosse encephalitis, an arboviral disease that is most commonly seen in the mid-Atlantic and midwestern areas of the United States (27, 28). This vector-borne virus is maintained in nature in sylvatic cycles within small mammal hosts such as chipmunks, squirrels, and field mice (29–31). Here, we demonstrate that the SGE of female *A. triseriatus* mosquitoes exhibits a strong anti-proliferative activity on murine and guinea pig splenocytes. This activity is characterized by cytokine suppression and induction of apoptosis. No effect was found in human lymphocytes or lymphoid cell lines.

## 2 Methods

### 2.1 Salivary gland dissections

The *Aedes triseriatus* colony used in this study was initially established from larvae identified and collected in used tires in Waterford, CT, USA. The strain was kindly donated by Dr. Doug E. Brackney (The Connecticut Agricultural Experiment Station, New Haven, CT). *Aedes albopictus* (New Jersey 99), *Anopheles gambiae* (G3), *An. stephensi* (Nijmegan), *Culex quinquefasciatus* (Hilo, Hawaii), and *Lutzomyia longipalpis* (Jacobina) were reared at the Laboratory for Malaria and Vector Research, NIAID, NIH under standard conditions (27°C, 80% humidity, and a 12-hr light/dark cycle). Salivary glands from adult female sugar-fed mosquitoes were dissected under a stereomicroscope and immediately placed into phosphate-buffered saline pH 7.4 (PBS) (Gibco, ThermoFisher Scientific). To obtain SGE, the dissected salivary glands were sonicated to disrupt salivary gland tissue (FisherBrand Model 120 Sonic Dismembrator). The homogenates obtained were then centrifuged at 12,500 x g for 5 minutes to remove debris. The supernatants were stored at -80°C until further use. For *A. triseriatus*, protein yields were highly consistent; on average, 20 salivary gland pairs in 20 μl of PBS produced 1.84 ± 0.11 mg/mL of protein, corresponding to 1.84 µg per pair, with a final protein concentration of 9.2 µg/mL per assay.

### 2.2 Animals

Eight-to 12-week-old female BALB/c mice (strain code 028) were purchased from Charles River Laboratories (Wilmington, MA, USA). The vendor ensured that animals were free of the following pathogens: Helicobacter, MHV, MNV, MPV, and Parvovirus. All experimental procedures were conducted in accordance with an approved NIAID-NIH animal study protocol (approval ID: ASP-LMVR3) and were overseen by the NIAID Animal Care and Use Committee (ACUC). Mice and guinea pigs (Hartford) were housed under standard laboratory conditions and were provided with *ad libitum* food and water. Spleens from rats (Sprague Dawley) and rabbits (New Zealand White) were kindly donated by the Twinbrook Animal Facility, Comparative Medical Branch, NIAID, and Animal Studies Unit, Laboratory of Malaria Immunology & Vaccinology, NIAID, respectively.

### 2.3 Splenocyte isolation

Spleens were aseptically harvested from mice or other mammals and placed into Roswell Park Memorial Institute (RPMI) 1640 Medium (Gibco, ThermoFisher Scientific) supplemented with 10% fetal bovine serum (FBS) (Gibco, ThermoFisher Scientific) and 1% antibiotic-antimycotic solution (Gibco, ThermoFisher Scientific). Excised spleen tissue was mechanically disrupted by gently mashing the tissue through a 40-µm cell strainer (Falcon). Following tissue maceration, cold PBS was used to facilitate cell passage through the strainer more effectively. The cell suspension was then centrifuged at 445 g for 5 minutes at 4°C, and the supernatant was discarded. The cell pellet was resuspended in 1 mL of ACK Lysing Buffer (Gibco, ThermoFisher Scientific) and incubated for 1–3 minutes to remove red blood cells. Following the lysis step, the cell suspension was washed with cold PBS twice and the pellet was resuspended in RPMI 1640 Medium (Gibco, ThermoFisher Scientific) for downstream use.

### 2.4 Human lymphocytes isolation and culture

Whole blood was collected from medication free donors (Department of Transfusion Medicine, NIH Clinical Center) on the NCI IRB approved NIH protocol 99-CC-0168, “Collection and Distribution of Blood Components from Healthy Donors for *In Vitro* Research Use”. The EasyStep Direct Human Total Lymphocyte Isolation Kit (STEMCELL Technologies) was used to isolate total lymphocytes through immunomagnetic negative selection. After isolation, cells were seeded in 96-well plates at 200,000 cells per well in 200 µL of RPMI supplemented with 10% FBS (Gibco, ThermoFisher Scientific) and 1% antibiotic-antimycotic solution (Gibco, ThermoFisher Scientific).

### 2.5 Cell lines

All cell lines utilized in this study were maintained under controlled conditions at 37°C in an incubator with 5% CO^2^ and humidity. Jurkat, Clone E6-1 (ATCC, TIB-152) and Raji (ATCC, CCL-86) were cultured in RPMI 1640 Medium (Gibco, ThermoFisher Scientific) supplemented with FBS (Gibco, ThermoFisher Scientific) and 1% antibiotic-antimycotic solution (Gibco, ThermoFisher Scientific). Human microvascular endothelial cells (HMEC-1, ATCC, CRL-3243) were cultured in complete growth medium according to ATCC guidelines. Keratinocytes (PCS-200-011) and Fibroblasts (PCS-201-010) were obtained from ATCC and maintained according to ATCC guidelines. All cell cultures were routinely monitored for contamination, and all experiments were performed within a limited passage range to ensure the reproducibility of experiments.

### 2.6 Metabolic activity assay using AlamarBlue

Metabolic assays were carried out using the primary cells and cell lines mentioned above. For all metabolic assays, isolated cells were incubated with SGE for 15 minutes at 37°C under standard conditions (humidified atmosphere with 5% CO_2_). Following this incubation period, cells were stimulated to proliferate and were again cultured in RPMI medium supplemented with 10% FBS (Gibco, ThermoFisher Scientific) and 1% antibiotic-antimycotic solution (Gibco, ThermoFisher Scientific) for 48–96 hours. All cells were seeded in 96-well plates at a density of 200,000 cells per well in 200 µL of RPMI. Twenty-four hours before metabolic activity measurements, 20 µL of AlamarBlue Cell Viability Reagent (ThermoFisher Scientific) were added to each well, and the plates were incubated under standard conditions and protected from light. After 24 hours, the absorbance was measured using a VersaMax Microplate Reader (Molecular Devices) at 570 nm, with 600 nm as the reference wavelength. The reduction of AlamarBlue was calculated by first subtracting the background absorbance (absorbance in wells with media only) and then using the molar extinction coefficients of oxidized and reduced AlamarBlue, as specified in the AlamarBlue Cell Viability Assay Reagent instructions (ThermoFisher Scientific). The reductions of AlamarBlue reagent are used as a reference to determine a cell population’s relative metabolic activity. The specific stimulants used for these experiments were Concanavalin A (ConA) (Sigma-Aldrich), Dynabeads Human T-Activator CD3/CD28 beads (Gibco, ThermoFisher Scientific), Phytohemagglutinin (Invitrogen), Pokeweed, and Lipopolysaccharide (LPS, from Sigma).

### 2.7 Kinetic and dosage apoptosis assays

For kinetic and dosage apoptosis experiments, isolated splenocytes were exposed to varying concentrations of SGE or were incubated with SGE for different time points. In the kinetic assay, cells were treated with the equivalent of 1 pair of salivary glands per 200,000 cells for increasing time periods (0, 5, 15, 30, 60, 120, or 180 minutes). In the dosage assay, cells were exposed to increasing concentrations of SGE (equivalent to 0.0625, 0.125, 0.25, 0.5, or 1 pair of salivary glands per 200,000 cells) for a fixed incubation time of 180 minutes. After incubation, the cells were washed once with PBS (Gibco, ThermoFisher Scientific) and then resuspended in the assay buffer. The Abcam Apoptosis/Necrosis Detection Assay Kit was used to assess cell viability, necrosis, and apoptosis following the manufacturer’s protocol (Abcam, Cambridge, UK, ab176749). To evaluate cell viability, Cytocalcein 450 was detected using the violet channel (Ex/Em = 405/450 nm). To identify apoptotic cells, Apopxin Green Indictor was visualized in the FITC channel (Ex/Em = 490/525). Necrotic cells were detected using 7-aminoactinomycin D (7-AAD) in the PE channel (Ex/Em = 550/650). Samples were analyzed using a MACSQUANT 10 (Miltenyi), and compensation controls were applied using single-stained and unstained controls to ensure accurate gating of each population.

### 2.8 Lymphocyte-specific apoptosis assays

To determine specific subsets of lymphocytes that undergo apoptosis, cells were treated with 1 pair of salivary glands per 200,000 cells and were incubated for 4 hours. Following exposure, cells were washed with PBS (Gibco, ThermoFisher Scientific) and stained with monoclonal antibodies conjugated to a fluorochrome to identify lymphocyte populations of interest. Cells were resuspended in 1x PBS with 0.1% BSA and incubated with 5µL of the Super Bright Complete Staining Buffer (eBioscience, ThermoFisher Scientific, SB-4401-42) to prevent nonspecific antibody interactions.

An antibody cocktail containing anti-CD4 monoclonal antibody (RM4-5, FITC) (eBioScience, Thermofisher), PE rat anti-mouse CD8a (BD Pharmingen), and PE-Cy7 rat anti-mouse CD19 (BD Pharmingen) was added to each sample. Cells were incubated in the dark at 2–8°C for 30 minutes. Following this incubation, cells were washed and resuspended in Annexin V binding buffer (10 mM HEPES, 140 mM NaCl, and 2.5 mM CaCl_2_, pH 7.4). To identify apoptotic cells, Annexin conjugated to Alexa Fluor 647 (Invitrogen, ThermoFisher) was added to each sample, followed by a 15-minute incubation at room temperature in the dark. Immediately after incubation, cells were analyzed using flow cytometry with a MACSQUANT 10 (Miltenyi). Compensation controls were established using single-stained, unstained controls, and compensation beads (UltraComp eBeads, Invitrogen) for fluorochrome overlap. FlowJo v10 software was utilized for analysis. Apoptosis was also evaluated by immunoblotting. Under the same treatment conditions, whole-cell extracts were lysed and run on SDS-PAGE gels. The proteins were transferred to PVDF membranes and then incubated with the indicated antibodies: anti-Caspase-3 and Cleaved Caspase-3 (Asp175) (Cell Signaling), and anti-β-actin as a loading control (Sigma).

### 2.9 Cytokine ELISA

Mouse IL-2, TNF-α, IL-1β, and INF-γ secretion were measured 72 hours after exposure to *Ae triseriatus* SGE. To measure cytokine production, Mouse Uncoated ELISA Kits were used to determine the concentration of these cytokines in the supernatant of spleen cell cultures (Thermo Fisher Scientific). First, Corning Costar 9018 ELISA plates were coated with 100 μL/well of the capture antibody (diluted 1:250) and incubated overnight at 4°C. After incubation, the wells were washed and aspirated three times with the washing buffer and then blocked with 200 μL of the ELISA diluent for one hour at room temperature. After blocking, 100 μL of each sample was added to each well. The plate was then left to incubate for two hours and subsequently washed 3–5 times. The detection antibody was then added to each well, and the plate was incubated at room temperature for one hour. Next, the plates were washed and aspirated, and 100 μL of Streptavidin-HRP was added to each well and incubated for 30 minutes. Following incubation, five washes were conducted, and 100 μL of the TMB solution was added to each well. After 15 minutes incubation, the absorbance of each well was measured using a VersaMax Microplate Reader (Molecular Devices) at 450 nm, with 570 as the reference wavelength. A standard curve was generated by running dilutions of recombinant mouse cytokines alongside the supernatant samples.

## 3 Results

### 3.1 Salivary gland extracts from *Aedes* mosquitoes inhibit the metabolic activity of murine splenic lymphocytes

**Figure 1:**
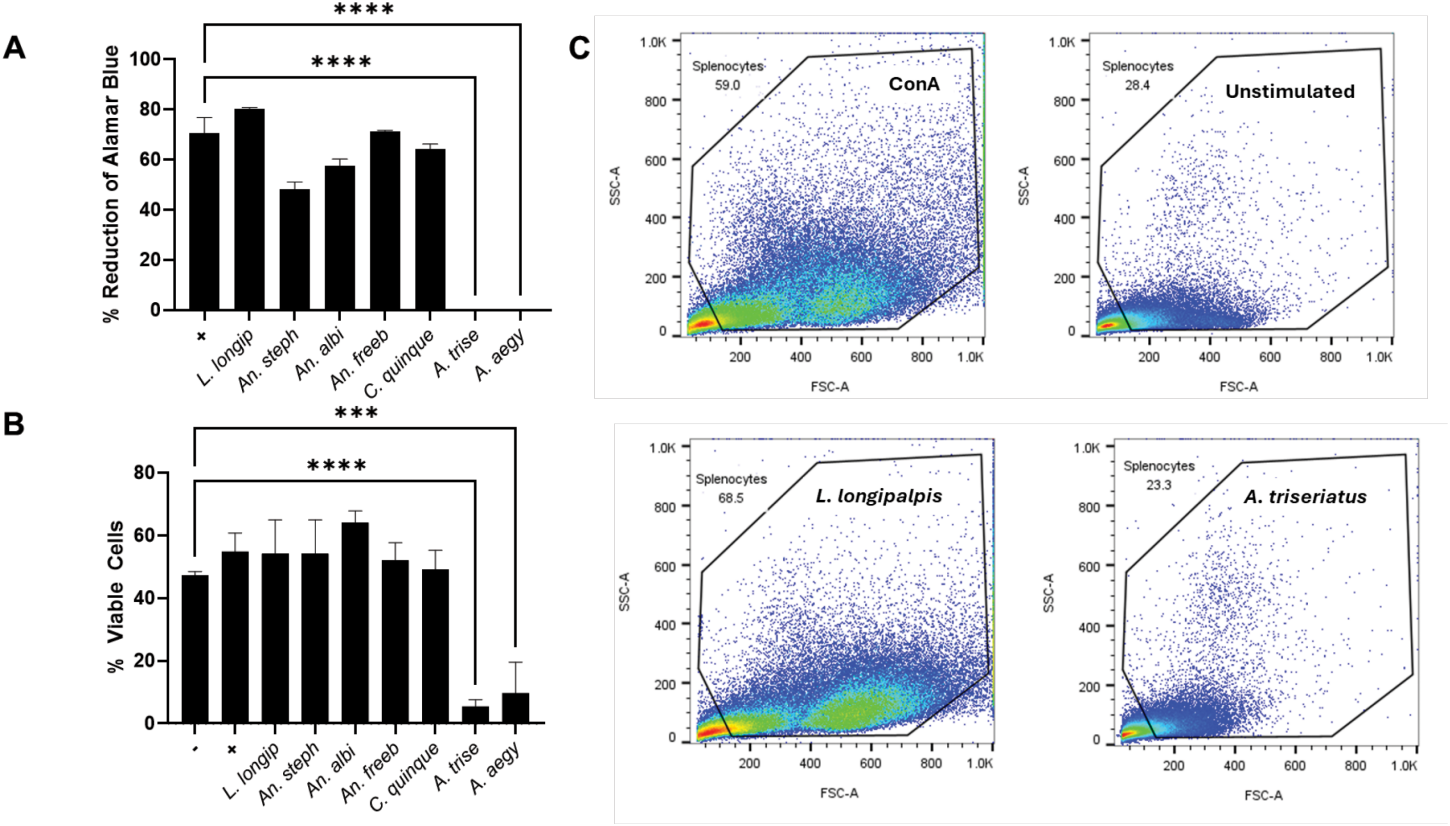
Salivary gland extracts from *Aedes* mosquitoes suppress splenocyte metabolic activity and viability. **(A)** Murine splenocytes (200,000 cells/well) were treated with SGE (4 pairs/well for 15 minutes) from *L. longipalpis*, *An. stephensi*, *An. albimanus*, *An. freeborni*, *C . quinquefasciatus*, *A . triseriatus*, and *A . aegypti*, followed by stimulation with ConA (1 µg/mL). Metabolic activity was quantified 96 hours post-stimulation using the AlamarBlue assay. **(B)** Viability of splenocytes treated under identical conditions was assessed at 72 hours by flow cytometry following live/dead staining. **(C)** Representative flow cytometry plots display FSC-A vs. SSC-A gating, comparing ConA-stimulated controls to cells treated with *Lu. longipalpis* or *A triseriatus* SGE. The same gating strategy was applied consistently across all samples. Data represent mean (n =3) ± SD. Statistical significance was determined by one-way ANOVA; ***p = 0.0001, ****p < 0.0001.

Previous reports have demonstrated the immunomodulatory and antiproliferative effects of *A. aegypti* SGE on murine lymphocytes *in vitro* (13, 25, 26). To investigate whether this property is conserved in other hematophagous vectors, we tested SGEs from *A. triseriatus*, *C. quinquefasciatus, Anopheles*, and *Lutzomyia longipalpis* species on murine splenocytes. Cells stimulated with ConA in the presence of SGE of *A. aegypti* and *A. triseriatus* significantly reduced metabolic activity relative to the stimulated control (media alone). No significant effect on proliferation inhibition was observed in cells treated with SGE from the other species (**Figure 1A**). To further characterize this activity, we assessed cell viability by flow cytometry. Splenocytes exposed to *A. aegypti* and *A. triseriatus* SGEs exhibited a reduced frequency of viable cells compared to controls (**Figure 1B**). Notably, their FSC/SSC profile resembled that of unstimulated cells. In contrast, splenocytes exposed to SGE from *Culex*, *Anopheles*, or *Lutzomyia* maintained a profile similar to that of stimulated cells with no SGE exposure. Comparative analysis of *A. triseriatus* and *Lu. longipalpis* (**Figure 1C**) illustrates this effect. These findings demonstrate that SGE from *A. triseriatus* mosquitoes can actively suppress murine splenocyte metabolic activity, potentially through cytotoxic mechanisms that can kill murine splenocytes, similar to the mechanism proposed for *A. aegypti* SGE (26).

### 3.2 *Aedes triseriatus* SGE induces apoptosis in a time and dose-dependent manner

Apoptosis is a fundamental mechanism of programmed cell death, orchestrated by tightly regulated intrinsic and extrinsic pathways. Given that exposure to *A. triseriatus* SGE reduced murine splenocyte viability, we examined whether this effect is mediated by apoptosis. To investigate this, splenocytes were treated with increasing concentrations of *A. triseriatus* SGE or incubated for varying time points. Apoptotic cell death was assessed by flow cytometry using apopxin to detect early apoptotic events, 7-AAD to assess membrane permeability during late-stage apoptosis, and CytoCalcein to evaluate intracellular esterase activity and metabolic integrity. Cells retaining high CytoCalcein fluorescence (Cytohigh or Cytomed) were considered metabolically active and viable, but these populations reflect different stages of response. Cytohigh cells represent full viability, while Cytomed cells indicate an early transition toward reduced viability.

**Figure 2:**
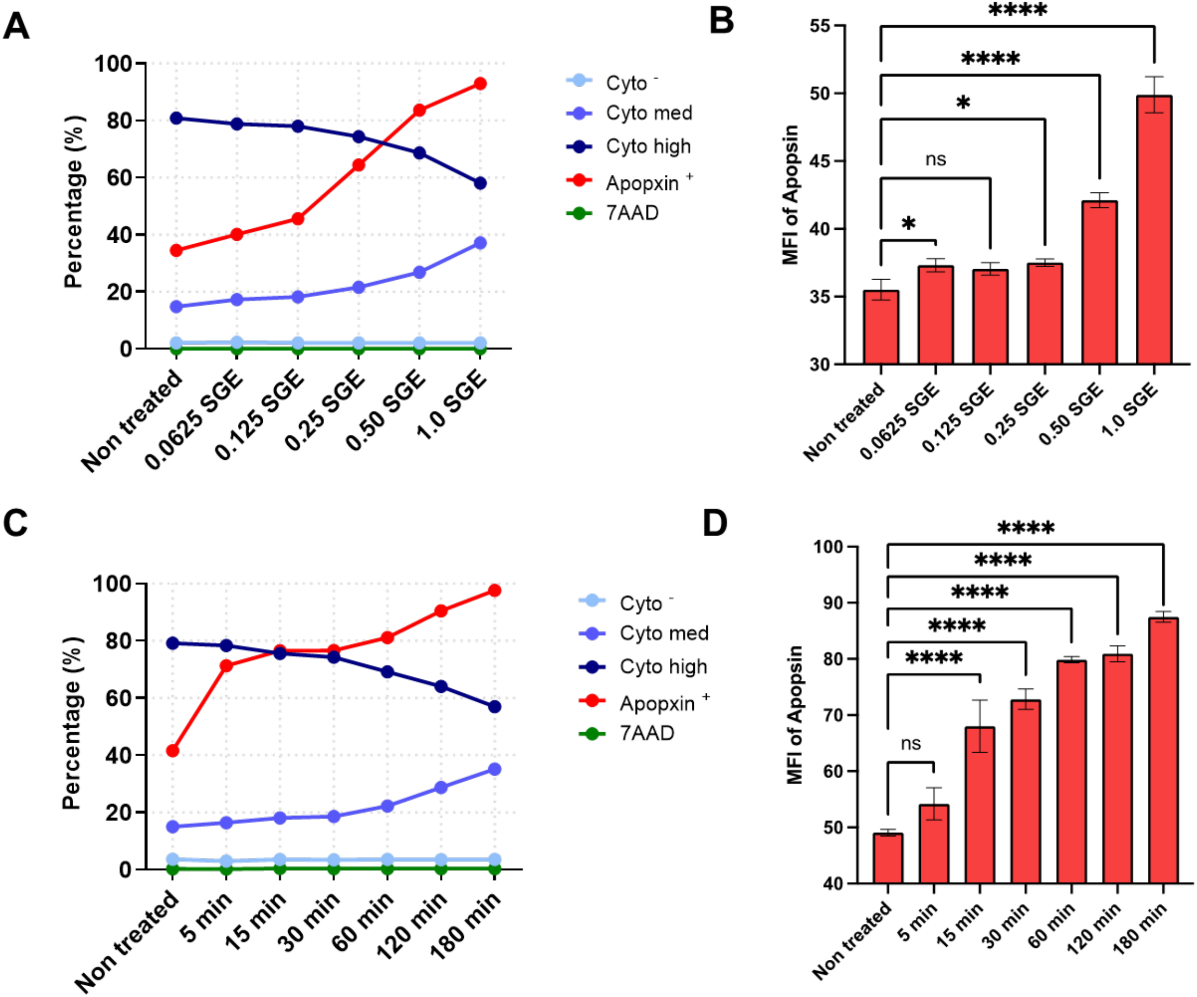
*Aedes triseriatus* salivary gland extract induces dose- and time-dependent apoptotic signaling in murine splenocytes. **(A, B)** Splenocytes were treated with increasing concentrations of *A. triseriatus* SGE (0.0625 to 1 gland pair/200,000 cells) for 3 hours. Cells were stained with an apoptosis/necrosis detection kit and analyzed by flow cytometry. Shown are the percentages of apopxin^+^, 7-AAD^+^, and Cytohigh^+^ cells **(A)**, and apopxin mean fluorescence intensity (MFI) **(B)**. For kinetic analysis, splenocytes were exposed to a fixed dose of SGE (1 gland pair/200,000 cells) for 5 to 180 minutes. Percentages of apopxin^+^, 7-AAD^+,^ and Cytohigh^+^ cells are shown **(C)**, along with apopxin MFI over time **(D)**. Data represents SD (n =3). Statistical analysis was performed using ordinary one-way ANOVA. *p = 0.0409; ****p < 0.0001.

Cells treated with 0.0625 to 1.0 gland pair equivalents of SGE showed a dose-dependent increase in apoptosis (**Figure 2A**). Untreated splenocytes had 34% apopxin^+^ cells, while those exposed to the highest dose (1 pair) for 3 hours reached 92.6%. This also caused a significant drop in Cytohigh+ cells from 80.8% to 59.1%, indicating loss of esterase activity and early dysfunction, with more Cytomed^+^ cells, showing early transition to reduced viability stage despite these changes, the frequency of 7-AAD^+^ cells remained unchanged across all SGE concentrations, suggesting that cells were undergoing early-stage apoptosis without nuclear membrane compromise. A dose-dependent increase in apopxin mean fluorescence intensity (MFI) was observed with increasing concentrations of SGE (**Figure 2B**), indicating more phosphatidylserine exposure and activation of apoptotic signaling pathways.

To investigate the kinetics of this response, splenocytes were treated with a fixed dose of SGE and analyzed over time. As shown in **Figure 2C**, the percentage of apopxin+ cells increased over time, while Cytohigh^+^ cell frequency declined from 79.1% in controls to 56.9% after 180 minutes, reflecting apoptotic progression death. Similarly, apopxin MFI increased as early as 15 minutes post-exposure and continued to rise over time, indicating rapid onset and amplification of apoptotic signaling (**Figure 2D**). Consistent with the dose-response findings, 7-AAD staining did not significantly increase during the early phases of exposure (**Figure 2C**), indicating an absence of late apoptotic or necrotic features within the first three hours. However, prior studies and additional time-course data suggest that 7-AAD^+^ cell frequency begins to rise after approximately five hours of exposure (25), marking a potential shift toward late-stage apoptosis or secondary necrosis. Together, these results demonstrate that *A. triseriatus* SGE induces rapid, dose- and time-dependent apoptotic cell death in murine splenocytes, characterized by early phosphatidylserine exposure and metabolic decline, with minimal evidence of nuclear membrane disruption during the early exposure window.

### 3.3 Murine lymphocytes undergo apoptosis following exposure to *A. triseriatus* SGE

Given the observed cytotoxic effects of *A. triseriatus* SGE on total splenocytes, we next investigated whether specific lymphoid populations were selectively affected. To assess this, splenocytes were treated with *A. triseriatus* SGE for four hours and analyzed by flow cytometry using Annexin V to detect phosphatidylserine exposure, a marker of early apoptosis.

**Figure 3:**
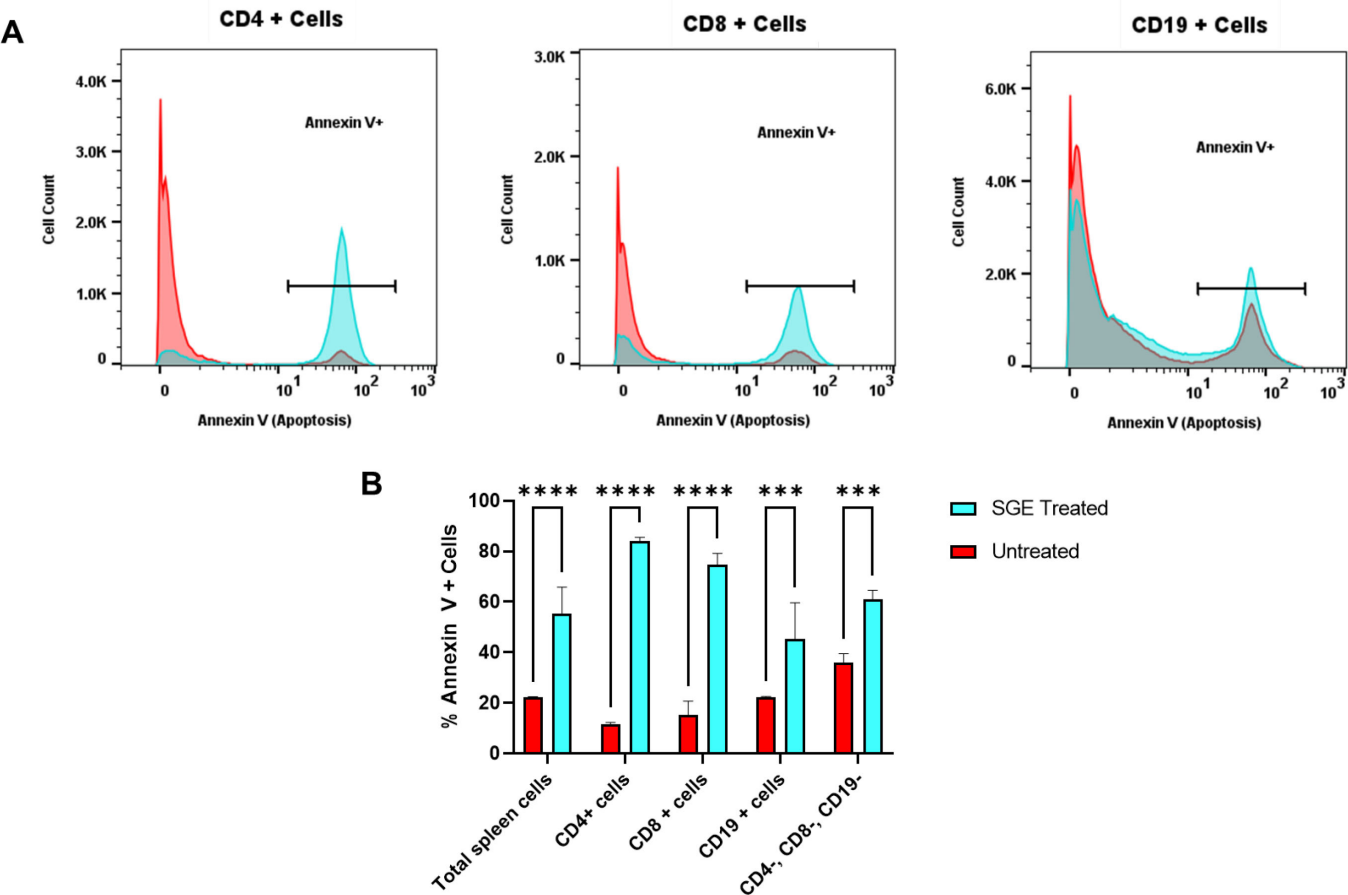
Lymphocytes susceptible to *A. triseriatus* SGE cytotoxicity. Murine spleen cells were incubated with *A. triseriatus* SGE (1 gland pair per 200,000 cells) for 4 hours. After incubation, cells were washed, stained with antibodies against CD4, CD8, and CD19, and subsequently labeled with Annexin V to assess apoptosis by flow cytometry. **(A)** Representative histogram illustrates an increase in Annexin V^+^ cells following SGE treatment. **(B)** Quantification of Annexin V^+^ cells shows a significant increase in apoptotic CD4^+^ T cells, CD8^+^ T cells, CD19^+^ B cells, and cells of CD4^–^, CD8^–^, and CD19 ^–^ populations after SGE exposure. Data are presented as mean ± SD (n =3). Statistical analysis was performed using ordinary one-way ANOVA. Significance levels: ***p = 0.0001; ****p < 0.0001.

All analyzed populations (CD4^+^, CD8^+^ and CD19^+^) of cells were found to have a significant increase in apoptotic cells after a four-hour exposure to *A. triseriatus* SGE (**Figure 3A**). The percentage of cells positive for Annexin V^+^, across total splenocytes, and the CD4^–^, CD8^–^ and CD19 ^–^ population revealed that CD4^+^ and CD8^+^ T cells have the most pronounced apoptotic response, while CD19^+^ B cells showed a moderate but consistent increase (**Figure 3B**). This lymphocyte-specific susceptibility is consistent with previous reports of T cell apoptosis triggered by *A. aegypti* in murine spleen cell populations (26). An increase in the percentage of apoptotic CD4^–^, CD8^–^ and CD19 ^–^ cells suggests that there could be another unidentified population of myeloid or lymphoid spleen cells that also undergo apoptosis in response to *A. triseriatus* SGE exposure.

### 3.4 Cytokine production is diminished in murine splenocytes exposed to *A. triseriatus* SGE

**Figure 4:**
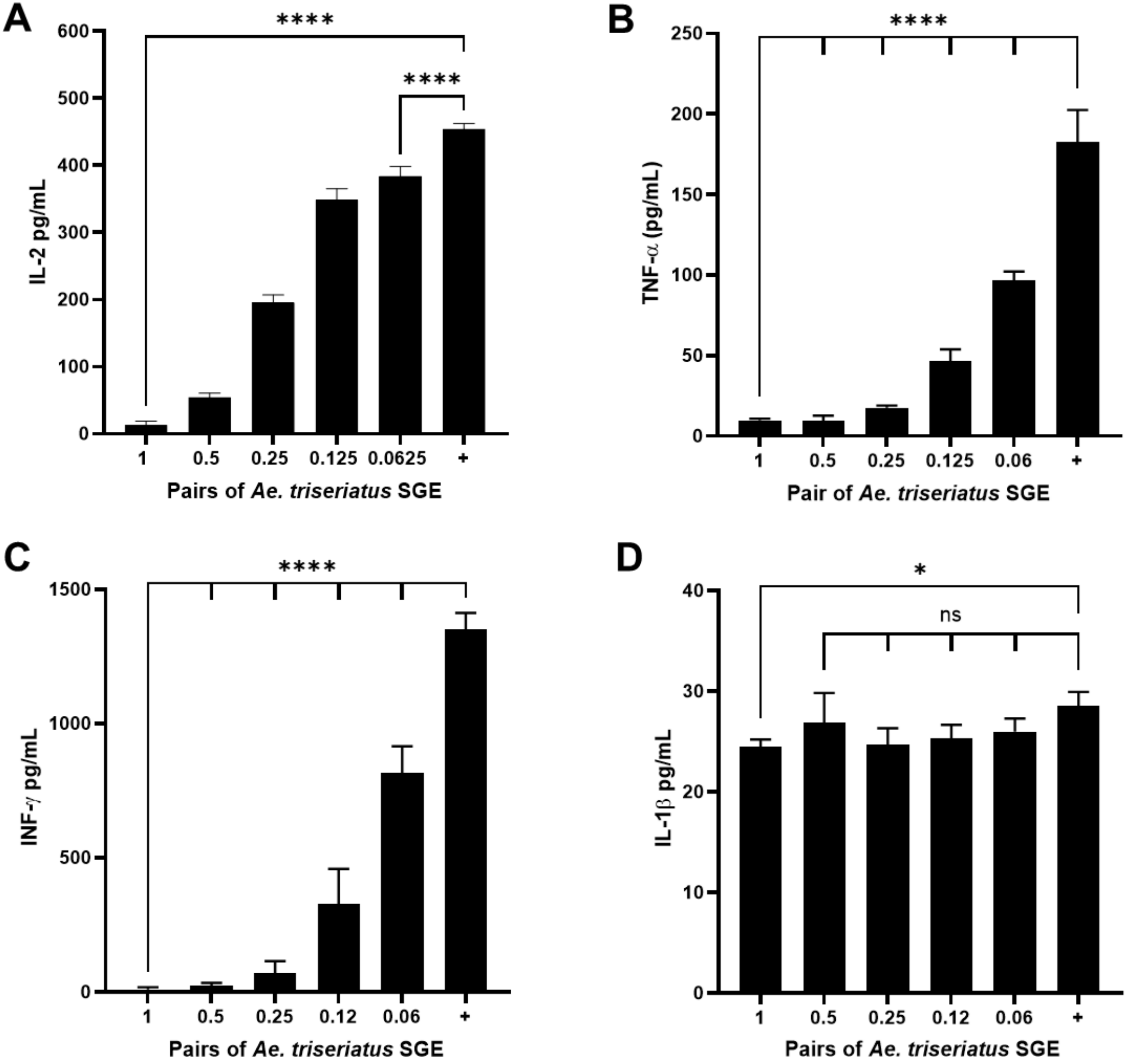
Dose-dependent modulation of cytokine secretion by *A . triseriatus* salivary gland extract. Murine splenocytes were exposed to decreasing concentrations of *A. triseriatus* SGE (1, 0.5, 0.25, 0.125, and 0.0625 gland pairs per 200,000 cells) and ConA. Supernatants were collected 72 hours post-treatment, and cytokine levels were quantified using ELISA. **(A–D)** Shown are the concentrations of IL-2, IFN-γ, IL-1β, and TNF-α. Data are presented as mean ± SD (n =3). Statistical analysis was performed using ordinary one-way ANOVA. Significance levels: *p = 0.0456; ****p < 0.0001; ns, not significant.

To assess the immunomodulatory potential of *A. triseriatus* SGE, we measured the secretion of IL-2, interferon-gamma (IFN-γ), tumor necrosis factor-alpha (TNF-α), and interleukin-1β (IL-1β) by splenocytes after ConA stimulation. After 72 hours, cells treated with SGE showed a clear dose-dependent suppression of IL-2, TNF-α, and IFN-γ (**Figures 4B–C**). In contrast, IL-1β levels were only significantly reduced at the highest dose (1 pair), suggesting a more limited sensitivity to SGE at the evaluated time point (**Figure 4D**).

### 3.5 Cell metabolism rates in non-murine mammalian lymphocytes

**Figure 5:**
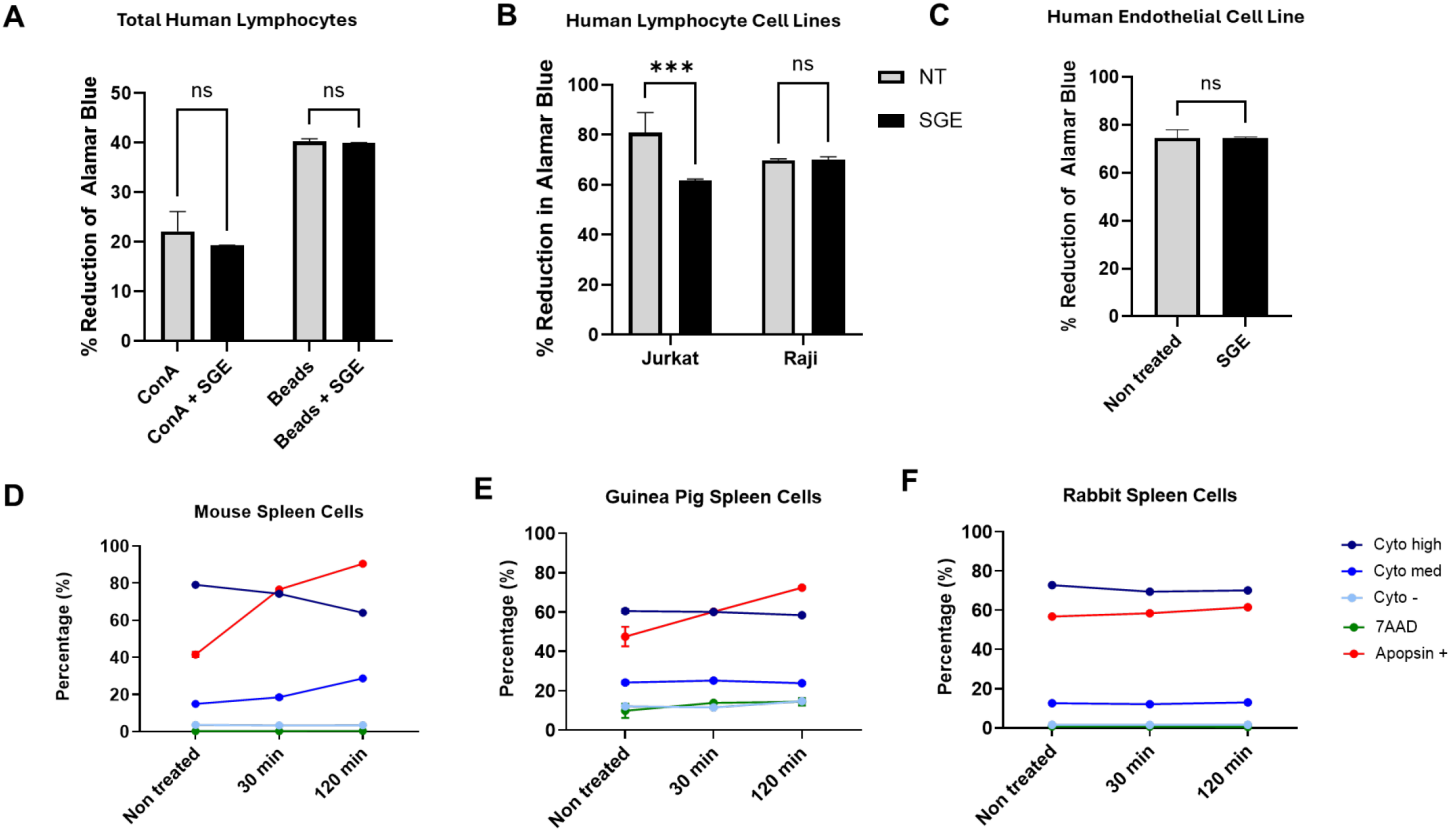
Immunomodulatory effects of *A triseriatus* SGE across species. **(A)** Human peripheral blood lymphocytes were exposed to *A . triseriatus* SGE (1 pair/200,000 cells) and stimulated with either ConA or anti-CD3/CD28 beads. **(B)** Human cell lines Jurkat (T-cell leukemia), Raji (B-cell lymphoma (40,000 cells/well), and **(C)** HMEC-1 (20,000 cells/well) were incubated with 1 pair of *A . triseriatus* SGE for 72 hours, and metabolism was evaluated using AlamarBlue. **(D–F)** Spleen cells were isolated from a mouse, guinea pig, and rabbit and treated with *A . triseritus* SGE (1 pair per 200,000 cells) for 30 or 120 minutes. After this incubation, cells were analyzed using flow cytometry. The percentages of cells positive for 7-AAD, Apopxin, or Cytocalcein (Cyto) are shown. Data are presented as mean ± SD. Statistical analysis was performed using ordinary one-way ANOVA, with significance levels as follows: ***p < 0.009; ns, not significant.

After characterizing a strong apoptotic response to *A. triseriatus* SGE in murine lymphocytes, we next investigated whether similar effects could be observed in lymphocytes or other cell types derived from non-murine mammalian hosts. We first evaluated the response of human lymphocytes by isolating total lymphocyte populations from healthy donors. Cells were exposed to *A. triseriatus* SGE and stimulated either with ConA or anti-CD3/CD28 beads. Metabolic activity was evaluated 72 hours later using the AlamarBlue assay. No significant reduction in metabolic activity was detected following SGE exposure, suggesting that human lymphocytes do not undergo apoptosis or loss of viability under these conditions (**Figure 5A**). We repeated the assay using two human lymphoid cell lines: Jurkat (derived from a T-cell leukemia) and Raji (derived from a B-cell lymphoma). Jurkat cells had a modest reduction in metabolic activity following SGE exposure, whereas Raji cells showed no significant change (**Figure 5B**). These results support the observation that *A. triseriatus* SGE does not strongly impair the viability of human lymphoid cells, even those with leukemic or lymphomatous backgrounds.

We next evaluated whether *A. triseriatus* SGE affects the viability of non-hematopoietic human cells by testing a human endothelial cell line, HMEC-1 (Human Microvascular Endothelial Cells-1). No significant difference was observed between SGE-treated and untreated HMEC-1 cells, indicating that endothelial viability is unaffected under these conditions (**Figure 5C**).

Finally, to determine whether SGE-induced apoptosis varies among other mammalian species, we examined splenocytes from guinea pig and rabbits. Cells were exposed to SGE and assessed for viability, apoptosis, and necrosis at 30- and 120-minute post-treatment using flow cytometry. Compared to murine splenocytes, both guinea pig and rabbit cells showed a reduced shift in the percentage of apoptotic cells following SGE exposure (**Figures 5D-F**), with a reduced decline in Cyto high cell frequency, and a limited number of cells transitioning to Cyto med or Cyto compared with non-treated cells. Together, these findings suggest that the pro-apoptotic effects of *A. triseriatus* SGE show a species-specific behavior, with a strong effect on murine splenocytes but not on human, rabbit, or guinea pig immune cells.

## 4 Discussion

Arboviruses are viruses transmitted by vectors, such as mosquitoes or ticks. Many of these viruses can cause disease in humans following transmission via the bite of an infected vector. Mosquito-borne viruses typically replicate in the skin at the initial site of injection, inducing cellular immune activation and recruitment, and subsequently hijack the recruited cells for further dissemination. This immune response is dominated by neutrophils, dendritic cells, and infiltrating monocytes. It has been suggested that these cells may be influenced by salivary gland proteins secreted by the mosquito, along with the virus, during a blood meal. Mosquito saliva is well known for its ability to modulate host immune responses (20, 32–37). Work by Cross et al. (1994) (38) demonstrated that SGEs from *A. aegypti* inhibited the proliferation of murine splenocytes stimulated with ConA, with a concurrent reduction in the production of IFN-γ and IL-2. Follow-up studies in OVA-TCR DO11 mouse splenocytes confirmed these immunosuppressive effects and revealed a dose-dependent response (26). Importantly, this activity was specific to female mosquito saliva and was abrogated by boiling or enzymatic digestion, indicating the presence of heat-labile protein factors (24). While the immunosuppressive effect of mosquito salivary secretion is well-documented in *A. aegypti*, it has not been previously investigated in *A. triseriatus*, the primary vector of La Crosse virus, which causes pediatric arbovirus encephalitis in the United States. Although *A. triseriatus* saliva is implicated in potentiating virus transmission (39), the molecule(s) responsible for this activity remain elusive thus far.

In this work, we describe the immunosuppressive activity of SGE from female *A. triseriatus* mosquitoes. We found a clear, dose-dependent inhibition of murine splenocyte proliferation following exposure to female SGE but absent in male SGEs (**Supplementary Figure 1A**). This sex and tissue specificity suggests the possible function of splenocyte proliferation inhibition in blood feeding and pathogen transmission. As with *A. aegypti*, this effect was abolished by heat treatment, further supporting the role of heat-sensitive salivary proteins in mediating the activity (**Supplementary Figure 1B**). The effect on the proliferation inhibition is independent of the mitogenic stimulus (**Supplementary Figure 2**). However, the molecule(s) responsible for this activity remain to be found in both species.

Our comparative assays revealed that SGEs from *C. quinquefasciatus*, *Anopheles* spp., and *L. longipalpis* flies did not elicit significant suppression of proliferation, as reported previously from another species (25, 26), while SGEs from *A. aegypti*, *A. triseriatus*, and *A. albopictus* (**Supplementary Figure 1D**) consistently inhibited splenocyte activity.

To explore the underlying mechanism, we assessed apoptosis and cell viability by flow cytometry. Even in the absence of mitogenic signals, increasing doses of SGE corresponding to up to 1 salivary gland pair equivalent led to a dose-dependent increase in Apopxin-positive cells and a concomitant reduction in viable Cytocalcein^+^ cells. Notably, we did not observe significant 7-AAD staining or caspase-3 activation (**Supplementary Figure 3**), suggesting that the observed loss of viability reflects early apoptosis rather than necrosis. Previous studies show that splenocyte death can occur without classical apoptotic markers, through pathways such as necroptosis, autophagy, kinase-mediated signaling, or mitochondrial dysfunction (40). However, a longer exposure may lead to necrosis effects, as have been observed in *A. aegypti* (25). Time-course analysis further confirmed this apoptotic response, as evidenced by increased Annexin V staining over time and concentration, indicating early exposure of phosphatidylserine. This apoptotic effect was not evenly distributed across lymphocyte subsets. Phenotypic characterization revealed that CD4^+^ and CD8^+^ T cells were more susceptible to apoptosis than CD19^+^ B cells, consistent with findings reported by Bizzarro et al. (2013) in *A. aegypti*, where apoptosis was mediated via caspase-3 and caspase-8 activation (26).

These immunosuppressive effects extended to the cytokine response as well. Cytokine production is an important indicator of lymphocyte activation and function. Interleukin-2 (IL-2) plays a central role in promoting T cell expansion and has previously been shown to be suppressed by salivary proteins from *Ixodes scapularis*, resulting in reduced T cell proliferation (41). In our assays, SGE from *A. triseriatus* inhibited the secretion of cytokines IL-2, TNF-α, and IFN-γ. While IL-1β levels were only slightly affected at the highest concentration tested, the overall cytokine profile suggests a broad immunosuppressive effect aligned with inhibition of proliferation and induction of apoptosis. Interestingly, this immunosuppressive effect did not extend to human cells. As we observed, using primary lymphocytes from healthy donors and highly proliferative lymphoid cell lines (Raji, Jurkat), Jurkat cells showed a slight reduction in proliferation, compared with our experiments using the SGE from *A. triseriatus*. No changes in proliferation were observed in non-hematopoietic cells like HMEC-1 or skin-derived cells such as fibroblasts and keratinocytes, indicating that SGE did not influence the growth of these cell types (**Supplementary Figure 4**). With these results, we suggest that caution should be exercised when extrapolating mouse-based findings to human immune systems in the context of mosquito saliva.

La Crosse virus, transmitted by *A. triseriatus*, uses small mammals such as chipmunks and squirrels as its primary reservoirs (29–31). We also tested whether the pro-apoptotic effect was conserved across rodent species. Indeed, only murine and guinea pig lymphocytes displayed Apopxin positivity upon exposure, pointing to a possible evolutionary adaptation in salivary composition to modulate the immune responses of natural hosts, potentially facilitating viral replication or transmission. In summary, *A. triseriatus* SGE exerts potent immunosuppressive effects on murine lymphocytes, characterized by proliferation inhibition, induction of apoptosis, and downregulation of key cytokines. These effects appear to be both host- and species-specific, highlighting the importance of careful interpretation of murine data and raising the possibility that salivary proteins have evolved to favor arbovirus persistence in rodent reservoirs. The biological relevance of lymphocyte proliferation inhibition by *A. triseriatus* SGE in blood feeding and/or pathogen transmission needs to be investigated in more detail. Future work should be done to identify the specific salivary molecules that mediate these immunosuppressive responses. Biochemical fractionation, comparative proteomics across mosquito species, and experiments using knockout mosquitoes are promising strategies to isolate the active factors and understand how they work. Collectively, these findings reveal new insights into the host- and genus-specific immunosuppressive strategies of mosquito saliva and suggest an evolutionary role for salivary proteins in shaping vector-host-pathogen interaction.

## Data Availability

The original contributions presented in the study are included in the article/**Supplementary Material**. Further inquiries can be directed to the corresponding author.
