# Supplementary material for: Immunosuppressive impact of Aedes triseriatus salivary gland extract on lymphocyte biology
Source: Front Immunol. 2026 Jan 27;16:1701532. doi: 10.3389/fimmu.2025.1701532 (PMC12886411; doi:10.3389/fimmu.2025.1701532)
Supplement: Supplementary file 1 [file DataSheet1.docx]

Supplementary Material


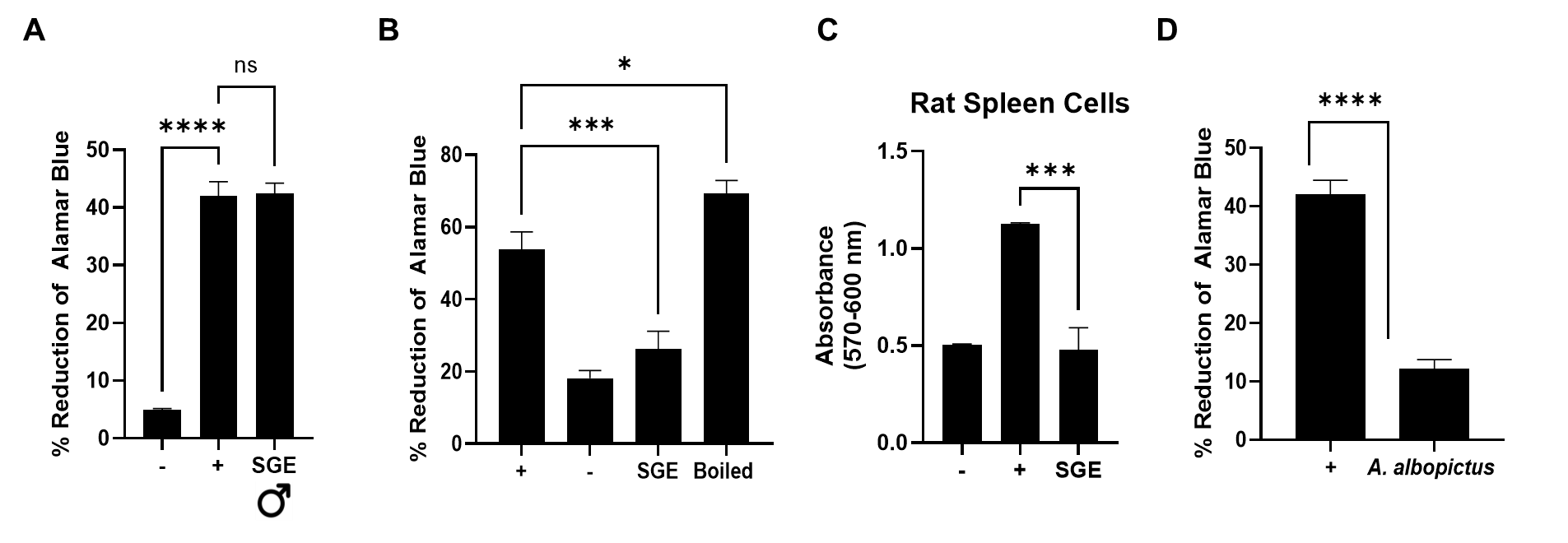


Supplementary Figure 1: Mosquito SGE inhibits splenocyte proliferation in mice and rats. Mouse splenocytes were incubated with *A. triseriatus* SGE from males (A) or heat-inactivated (boiled) females (B) at a concentration of 1 salivary gland pair per 200,000 cells. Cells were then stimulated with ConA (1 µg/mL). The same procedure was repeated with rat splenocytes (C) or SGE from *A. albopictus*. Symbols indicate treatment conditions as follows: ‘–’ indicates unstimulated cells (no ConA), ‘+’ indicates cells stimulated with ConA alone. Cell proliferation was measured by AlamarBlue reduction after 72 hours. Data are shown as mean ± SD (n = 3). Statistical significance was determined by one-way ANOVA (*p = 0.0107; ***p < 0.001; ****p < 0.0001; ns = not significant).

Supplementary Figure 2*: Aedes triseriatus* SGE inhibits murine splenocyte proliferation independently of the mitogenic stimulus. Murine splenocytes were exposed to SGE from female *A. triseriatus* (1 pair/200,000 cells) and stimulated with LPS (1 µg/mL), ConA (1 µg/mL), PHA (1X), or pokeweed mitogen (2.5 µg/mL). Metabolic activity was assessed 96 hours later using the AlamarBlue assay. Data represent mean ± SD. Statistical analysis was performed using ordinary one-way ANOVA with multiple comparisons among all four groups. Significance levels: ***p < 0.002.

**
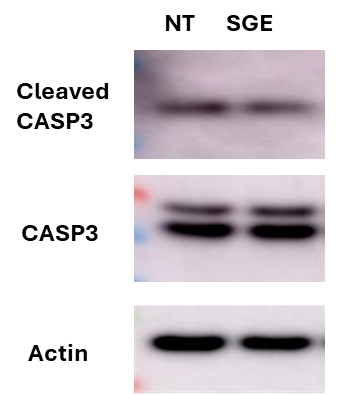
**

**Supplementary Figure 3**: **Caspase-3 analysis in splenocytes exposed to *Aedes triseriatus* salivary gland extract.** Splenocytes were incubated for 5 h with 1/2 salivary gland pair equivalent *of A. triseriatus SGE*. Western blotting was performed using anti–caspase-3 and anti–actin antibodies. No detectable changes were observed in full-length or cleaved caspase-3 after SGE treatment, and actin levels remained stable across samples.

**Supplementary Figure 4:** Proliferation of fibroblasts and keratinocytes treated with *A. triseriatus* salivary gland extract. (A) Fibroblasts (5,000 cells/well) and (B) keratinocytes (20,000 cells/well) were incubated for 72 h with 1 salivary gland pair equivalent of *A. triseriatus* SGE. Cell proliferation was evaluated using the Alamar Blue assay. Data were analyzed using an unpaired t-test; ns indicates no significant difference compared with untreated controls.
